# Improvement in Detection Limit for Lateral Flow Assay of Biomacromolecules by Test-Zone Pre-enrichment

**DOI:** 10.1038/s41598-020-66456-1

**Published:** 2020-06-15

**Authors:** Yi Zhang, Xiao Liu, Lingling Wang, Hanjie Yang, Xiaoxiao Zhang, Chenglong Zhu, Wenlong Wang, Lijing Yan, Bowei Li

**Affiliations:** 1 State Key Laboratory of Food Science and Technology, International Joint Laboratory on Food Safety, Collaborative innovation center of food safety and quality control in Jiangsu Province, Institute of Analytical Food Safety, School of Food Science and Technology, Jiangnan University, Wuxi, 214122 PR China; 2 Jiangnan University Hospital, Wuxi, 214122 PR China; 3 CAS Key Laboratory of Coastal Environmental Processes and Ecological Remediation, Research Center for Coastal Environmental Engineering and Technology, Yantai Institute of Coastal Zone Research, Chinese Academy of Sciences, Yantai, 264003 PR China

**Keywords:** Analytical chemistry, Biochemistry

## Abstract

Lateral flow assay (LFA) is one of the most prevalent commercially available techniques for point-of-care tests due to its simplicity, celerity, low cost and robust operation. However, conventional colorimetric LFAs have inferior limits of detection (LODs) compared to sophisticated laboratory-based assays. Here, we report a simple strategy of test-zone pre-enrichment to improve the LOD of LFA by loading samples before the conjugate pad assembly. The developed method enables visual LODs of miR-210 mimic and human chorionic gonadotropin protein, to be improved by 10–100 fold compared with a conventional LFA setup without introducing any additional instrument and reagent except for phosphate running buffer, while no obvious difference occurred for Aflatoxin B1 (AFB1). It takes about 6–8 min to enrich every 50 μL of sample diluted with phosphate running buffer, therefore we can get visual results within 20 min. We identified a parameter by modeling the entire process, the concentration of probe-analyte conjugate at test zone when signaling unit being loaded, to be important for the improvement of visual limit of detection. In addition, the test-zone pre-enrichment did not impair the selectivity when miR-210 mimic was adopted as target. Integrated with other optimization, amplification and modification of LFAs, the developed test-zone pre-enrichment method can be applied to further improve LOD of LFAs.

## Introduction

Lateral flow assay (LFA), a paper-based *in-situ* detection platform, has been widely used in diverse fields such as biomedicine, environmental health, quality control and food safety due to its rapid, inexpensive, portable measurements^1^. Basing on affinity interactions such as hybridization of oligonucleotide or aptamer-target, antibody-antigen or biotin-streptavidin, the signal of the test zone on the LFA test strip changes with the concentration of a particular analyte of interest when a liquid sample is applied^2,3^. There are two basic formats of LFA: sandwich format and competitive format. In addition, the competitive format could be designed in the following two ways: fixing the capture probe of target at test zone (competitive format I) or fixing the competitive analogue at test zone (competitive format II).

Generally, there are two strategies to load signaling unit in the conventional LFA. The first one is the direct sampling method with drying signaling unit on the conjugate pad fixed between the nitrocellulose (NC) membrane and the sample pad before sampling. This strategy is the most widely used and easily handled one and the results usually show significant concentration dependence. However, the limit of detection (LOD) is usually poor, which is the main drawback of this conventional LFA. The other strategy is the pre-mixing/pre-incubation sampling method with mixing signaling unit with sample solution to load together onto the sample pad^4^. Signaling unit is greatly diluted by sample solution in this strategy, resulting in a prolonged interaction between signaling unit and capture molecules at test zone and even false-positive results. Moreover, hundreds microliters of sample solution are always needed to be quantified precisely in the pre-mixing strategy, and that is not easy to achieve in resource-poor settings as the operator has to get micropipettes which usually cost hundreds of dollars.

There is an increasing need for the rapid *in-situ* detection of very low concentration of analyte such as microRNAs in the serum, which cannot be achieved using the conventional LFAs^5,6^. Alternative strategies to improve the sensitivity of LFA, such as temperature-humidity^7^ or fluidic control^8–11^, probe-based^12–14^ or enzyme-based signal enhancement^15,16^, and sample concentration or enrichment techniques^17–22^, have been reported. Methods to improve LFA sensitivity by enrichment or concentration can be roughly divided into on-strip pre-enrichment and off-strip pre-enrichment. The off-strip pre-enrichment methods include centrifuge filtration enrichment^23^, dialysis^5^, and interfacial enrichment^24^, while the on-strip pre-enrichment methods include isotachophoresis^17^, electromagnetic relocation^8^ and aqueous two-phase systems concentration^18^ *et al*. to control the mobility or location of analyte or signal unit. Whilst these sophisticated platforms achieve improved performance, they do involve either high-cost biochemical reagents such as enzymes^15,16^, external equipment such as electrophoresis apparatus^17,21^, complex fabrication^14^ or multiple-step operation^4,10^. Simplified and more cost-effective methods to improve the sensitivity of LFA are needed to be truly implemented for the rapid *in-situ* detection^25^.

Here, we report a simple strategy for test-zone pre-enrichment to improve the LOD of LFA. We changed the order of LFA strip assembling by installing conjugate pads after sampling rather than before. Theoretically, in sandwich format and competitive format I of LFA, the samples would be pre-enriched and captured at test zone with the proposed method, causing much higher concentration of probe-analyte conjugate at test zone than with direct sampling method. Therefore, higher sensitivity could be obtained due to the higher capturing rate of DP-AuNPs. We applied this strategy with miR-210 mimic, human chorionic gonadotropin (HCG) protein and Aflatoxin B1 (AFB1) as model targets. The LODs of miR-210 mimic were improved by 10–100 fold compared with the conventional LFAs, and those of HCG were improved by 10 fold. However, with competitive formats II, there are no obvious differences occurred. The method developed here did not involve any amplification reagent and instrument. We also modeled the entire process to identify parameters important for the improvement of visual LODs. The LOD could be further decreased via increasing the enriching volume, while the increase of sampling volume does not result in obvious improvement of LOD in conventional LFAs.

## Results and discussion

### Workflow of sampling methods

**Figure 1:**
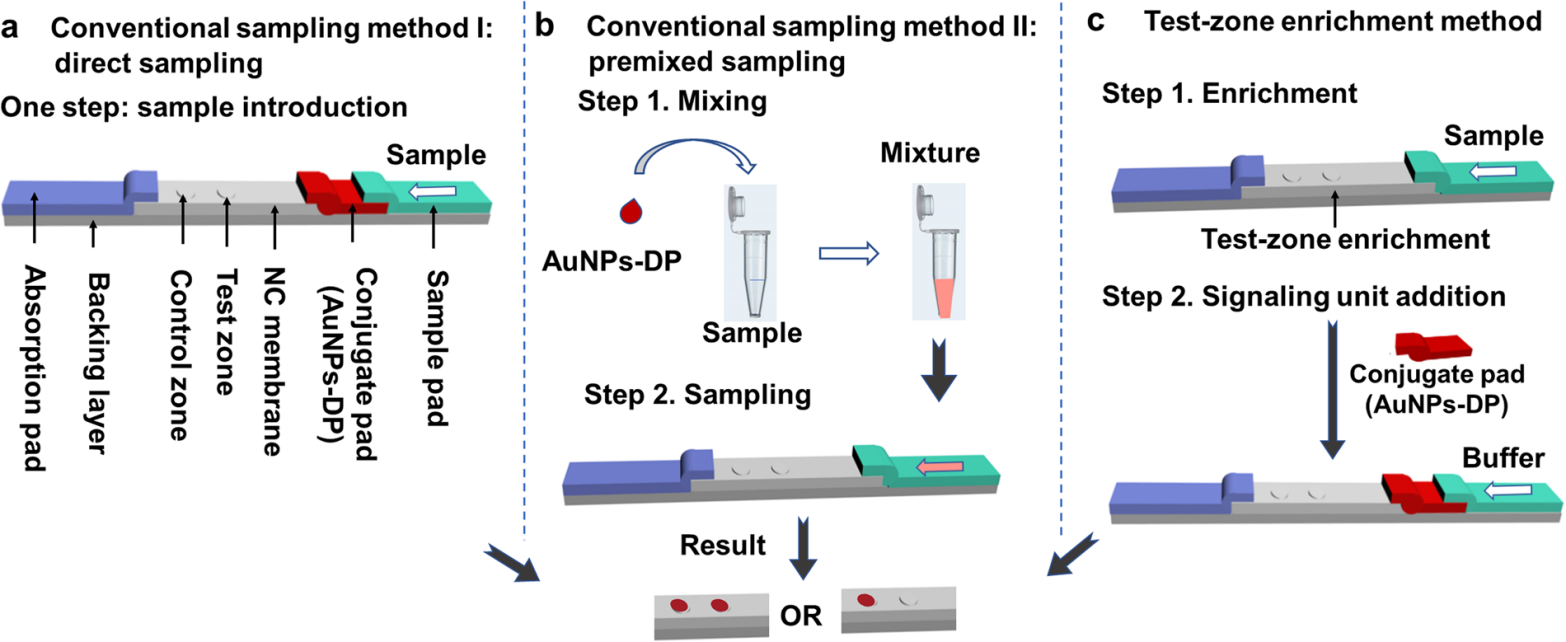
Schematic illustration of the sampling approach. (The structure of strips were initially drawn in Autodesk 3ds Max 2009 64-bit and then organized in Microsoft PowerPoint.).

The overall workflow of direct sampling, premixed sampling and test-zone pre-enrichment sampling methods is illustrated in Fig. 1. With the direct sampling method, the conjugate pad with dry signaling unit is pre-fixed between the nitrocellulose (NC) membrane and the sample pad before sampling and the strips are ready to use. With the pre-mixed/pre-incubated sampling method, signaling unit is mixed with sample solution to load together onto the sample pad. While with test-zone pre-enrichment sampling, the order of LFA strip assembling is changed. Specifically, we assembled conjugate pads after sampling rather than before. Therefore, the samples are pre-enriched and react with capture probe at test zone before signaling unit is uploaded. After pre-enrichment, the concentration of test zone probe-analyte conjugate increased. And higher capturing rate of signaling unit could be achieved when the signaling unit dried on conjugate pad is uploaded with running buffer.

### Validation in miR-210 LFAs

We explored a sandwich-like format and two competitive formats for miR-210 detection to develop the test-zone pre-enrichment sampling method, compared the performance of different sampling methods and also studied the volume effect.

### Sandwich-like format

**Figure 2:**
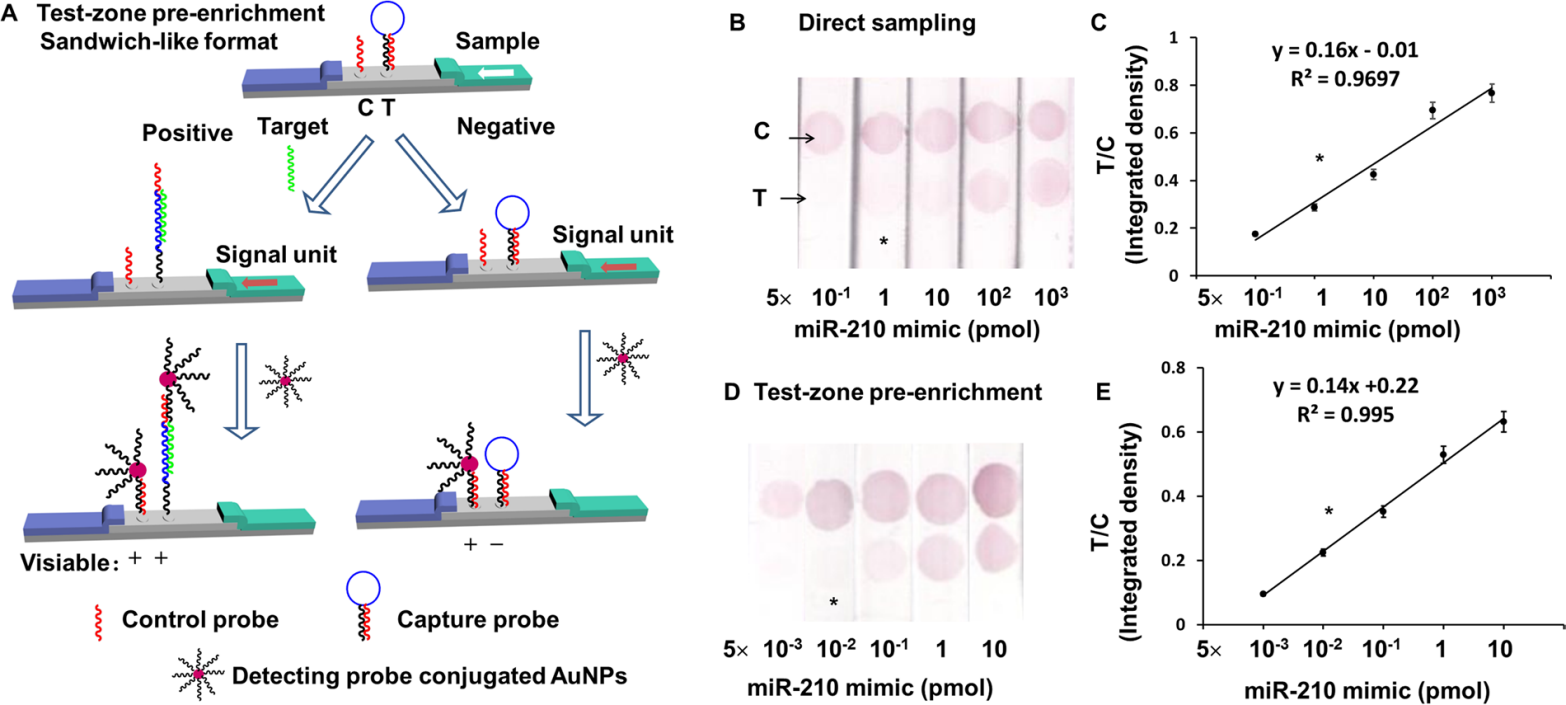
(**A**) Schematic depiction of the molecular setups for sandwich-like format LFAs for miR-210. (The structure of strips were initially drawn in Autodesk 3ds Max 2009 64-bit and then organized in Microsoft PowerPoint.) (**B**) Direct sampling and (**D**) test-zone pre-enrichment different amount of target (50 μL). (**C)** and (**E**) are integrated density ratio of test zone to control zone as functions of the target amount according to (**B**) and (**D**). Asterisk (*) indicates visual LOD. In the equation of calibration curves, y is the ratio of integrated intensity of test zone and control zone, while x is the log of miR-210 mimic concentration.

In sandwich-like format we used here, only when the target bound with the ring part of the molecular beacon (MB, as capture probe) at test zone and disassembled the self-binding at the stem of MB, the detecting probe on AuNPs could bind with the 3′ end of MB and showed a visible signal at test zone, while the probe at the control zone could capture AuNPs via binding to the detecting probe on the surface of AuNPs (Fig. 2A)^22^. Therefore the signal at test zone enhanced as the target concentration increased, while the signal at control zone kept unchanged high when there was an excess of DP-AuNPs.

**Figure 3:**
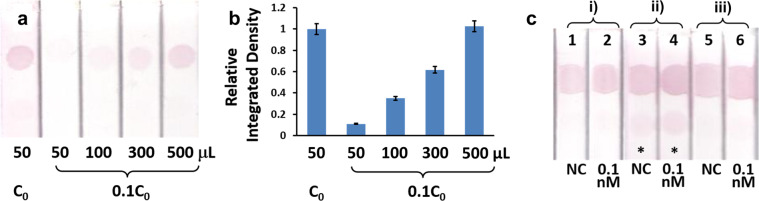
The enrichment effect of AuNPs-detecting probe conjugates on control zone in the absence of analyte detected by sandwich-like format. (**a**) Images of LFA strips. (**b**) Analysis results by ImageJ. C_0_ is the original concentration of AuNPs- detecting probe stock solution. (**c**) Validation of false positive by direct sampling a), pre-mixed sampling b) and test-zone pre-enrichment sampling of 50 μL samples c). Line 1, 3 and 5: negative control (NC), Line 2, 4 and 6: 0.1 nM analyte. Single asterisk indicates strip with visible test zone.

In order to check the feasibility of on-strip enrichment, 50 μL DP-AuNPs at concentration of C_0_ and 50–500 μL DP-AuNPs at concentration of 1/10 of C_0_ (diluted at 1:9 v/v by running buffer) were sampled to see the signal difference at control zone while no target was present in the sample solution (Fig. 3a,b). Interestingly, 500 μL DP-AuNPs (1/10 of C_0_) achieved 97–108% relative integrated density of 50 μL (C_0_) at control zone, indicating on-strip enrichment is reasonable and effective. However, in these tests working exactly as pre-mixing sampling method in nature, the LFA strips sampled with 50 μL DP-AuNPs (C_0_) and 500 μL DP-AuNPs (1/10 of C_0_) both showed obviously false-positive results at test zone. In addition, false-positive results still occurred at test zone when 50 μL buffer (negative control) or 0.1 nM target was tested with pre-mixing sampling method, while neither direct sampling nor test-zone pre-enrichment sampling gave obvious signal at test zone (Fig. 3c). Theoretically, nonspecific interaction between signaling unit (DP-AuNPs) and test zone probe is the primary reason for false-positive results. Under a certain amount of signaling unit, the ideal way to reduce nonspecific adsorption is to use a moderate concentration of AuNPs functionalized with a large number of antibodies or aptamers and blocked all extra sites by adsorbing protein molecules such as bovine serum albumin (BSA) on the surface, flowing through the detection area as fast as possible with the presence of detergent at certain concentration, but not low concentration of AuNPs highly diluted by sample solutions slowly passing the test zone^26,27^. The former one is exactly what happened in direct sampling method and test zone pre-enrichment sampling method. However, signaling unit is greatly diluted by sample solution in pre-mixing sampling strategy, causing blocking molecule BSA dissolving from the surface of AuNPs and thus some adsorption sites are exposed. In addition, the time of DP-AuNPs flowing through the test zone is greatly prolonged, resulting in further increasing of interaction between DP-AuNPs and test zone probe. Thus we did not further study the performance of pre-mixing sampling in detail.

Then the linear range and limit of detection (LOD) of direct sampling and test-zone pre-enrichment sampling were investigated (Fig. 2B–E). The visual LOD was 5 pmol by direct sampling 50 μL of sample, while it was only 50 fmol by test-zone pre-enrichment sampling 50 μL of sample with all the T/C ratios as function of sample concentration show 3–4 orders of magnitude linearity. The visual LOD was improved by 100 fold when test-zone pre-enrichment sampling method was applied. Thus test-zone pre-enrichment sampling can not only avoid false-positive results which happen occasionally in pre-mixing method, but achieve improved sensitivity compared with direct sampling. We are not claiming specifically about the absolute reduction in LOD of miR-210 that have been achieved or even surpassed with existing LFA tests that use different designs and optimized conditions. Significant optimization of the LFA must be completed to achieve the lowest possible LOD for a specific target in deed while we have not done much optimization here. Other methods to improve the LOD and the optimization of LFA can then be integrated with the developed test-zone pre-enrichment to further reduce the LOD.

As for a variety of sample pre-enrichment techniques, increase of sample amount, volume or duration inevitably leads to continuous increase in the signal before breakthrough^28–30^. On a similar principle, we assumed that in the sandwich-like format, the signal at test zone should vary constantly with the increase of sampling volume until the saturation of capture probes with all sampling methods of LFAs. To verify that assumption, we compared direct sampling as a classic sampling method with test-zone pre-enrichment sampling on sampling volume effect in sandwich-like format firstly. The increase in the pre-enriching volume up to 400 μL could lower down LOD further to 5 fmol, 10 fold improved compared with that achieved by pre-enriching 50 μL (Fig. S13). However, with direct sampling, no significant difference was observed by the naked eye or by image analysis software when loading 50, 100 or 400 μL of 10 nM miR-210 mimic, indicating this method worked at amount-independent but solely concentration-dependent mode (Fig. S4a,b). In addition, the response volume of direct sampling seemed much less than 50 μL because the signaling units flowed through the test and control zone at the forefront of the fluid in the first few minutes, explaining why there was no need to quantify sample volume in direct sampling (Fig. S4c).

In the pre-enrichment sampling method, no additional reagent except for running buffer and sample was needed throughout detection. The conjugate pad with dry DP-AuNPs was added after pre-enrichment of sample at test zone, therefore, the difference in operation time between direct sampling and pre-enrichment sampling mainly came from the step of enrichment. As it took about 6–8 min for enriching each 50 μL, 15–20 min for 100 μL and 60–90 min for 400 μL of sample depending on the viscosity of the sample solution and the room temperature, we researched the volume effect on enrichment up to 400 μL so that the enrichment could be accomplished within 1.5 h. The additional 6–20 min needed for enriching samples was acceptable as the common analysis time of other point-of-care detection is in the range of 10–30 min **(**Table S2**)**. Although we speculate that further increase in enriching volume may result in better sensitivity, enriching samples larger than 400 μL will take more than one hour to complete, which is not ideal enough as a point-of-care detection technology.

### Competitive format I

**Figure 4:**
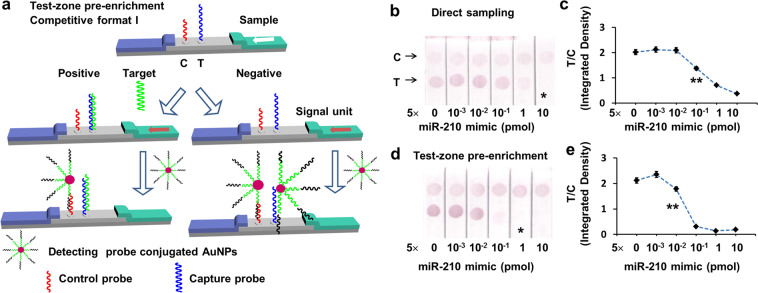
(**a**) Schematic depiction of the molecular setups for LFAs of competitive format I LFAs for miR-210. (The structure of strips were initially drawn in Autodesk 3ds Max 2009 64-bit and then organized in Microsoft PowerPoint.) (**b**) Direct sampling and (**d**) test-zone pre-enrichment different amount of target (50 μL). (**c**,**e**) are integrated density ratio of test zone to control zone as functions of the target amount according to (**b**,**d**). Asterisk (*) indicates the cutoff value as visual LOD, while double asterisks (**) indicate the LOD achieved by image analysis software.

In the competitive format I, capture probes were fixed at test zone. The signal at test zone decreased with the increase of target concentration in samples because the target competed with the detecting probe on AuNPs to bind with capture probe at test zone (Fig. 4a). The cutoff value, the threshold target concentration when the test zone turned colorless observed by the naked eye, was referred as the visual LOD, and semi-quantitative LODs were achieved by using a HP1139 scanner and analyzed by image analyzing software ImageJ. Direct sampling 50 μL of sample solutions offered the visual LOD of 10 pmol (Fig. 4b), and a semi-quantitative LOD of 100 fmol (Fig. 4c). In contrast, test-zone enriching 50 μL of sample solutions gave a visual LOD of 1 pmol (Fig. 4d) and a semi-quantitative LOD of 10 fmol (Fig. 4e). Both the visual LOD and the semi-quantitative LOD were improved by 10 fold, when test-zone enrichment was applied and the sample volumes were the same as those of direct sampling (50 μL). Further increase in the enriching volume was also proved to improve the LODs (Fig. S5).

The visual LODs got with the conventional pre-mixing method in the same format were also inferior to the developed test-zone pre-enrichment method (Fig. S6). In addition, the colors in the test zone and the control zone become obviously lighter with the increase of the sample volume from 50 μL to 100 μL, making it difficult to distinguish the chromogenic phenomena.

### Competitive format II

We also examined LFAs of competitive format II for miR-210 and Aflatoxin B1 (AFB1, as a model of small molecule analyte), respectively (Fig. S7). No obvious difference occurred between the pre-mixed sampling (recommended by the producer) and pre-enrichment sampling for AFB1, and so did the direct sampling and pre-enrichment sampling for miR-210, as we expected that there might be no enriching effect when the analog molecules of analyte were fixed on test-zone. Thus the pre-enrichment method we developed here only works well for the sandwich or sandwich-like format and competitive format I.

### Validation in HCG LFAs

**Figure 5:**
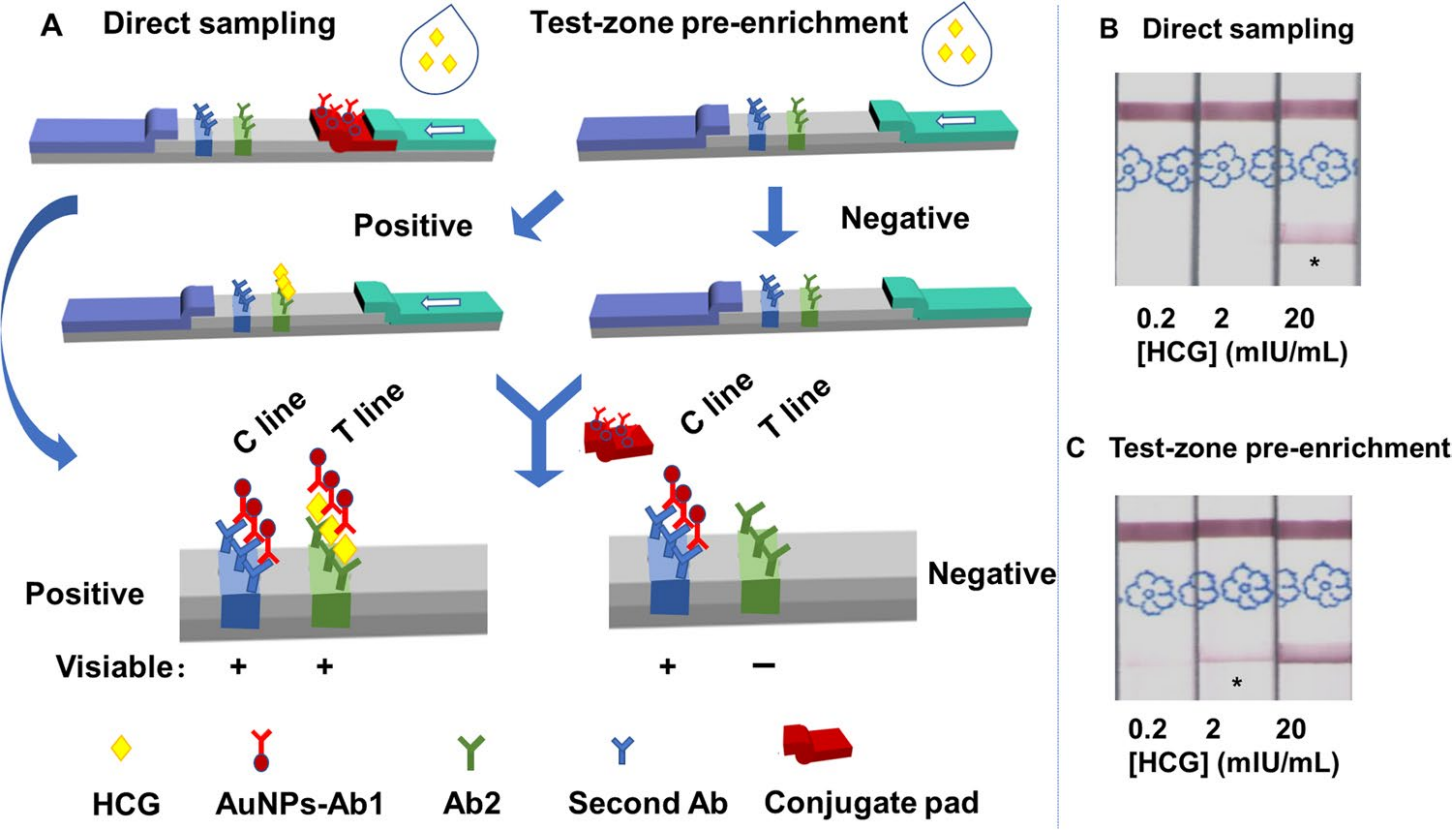
Images of HCG strips. Single asterisk (*) indicates the visual LOD. (The structure of strips were initially drawn in Autodesk 3ds Max 2009 64-bit and then organized in Microsoft PowerPoint.).

To verify the application of the proposed test-zone pre-enrichment method in the improvement of protein detection, we applied it to commercial HCG strips. The determination mechanism of HCG was classical sandwich format: the target (HCG) captured between the capturing probe (antibody 2 of HCG) and the signaling unit (AuNPs labeled antibody 1 of HCG) (Fig. 5A). The direct sampling method was accomplished according to the producer’s manual. As for the pre-enrichment method, the conjugated pad was removed from the strip firstly and inserted back after the loading of samples. The visual LOD of HCG was 20 mIU/ML (50 μL) detected with the direct sampling method (Fig. 5B), while the LODs achieved with pre-enrichment method were 2 mIU/mL (50 μL), 10 fold improved compared with that of direct sampling method (Fig. 5C**)**. The result indicates that the developed test-zone pre-enrichment method is applicable to the sensitivity improvement of protein analytes.

### Simulation and modeling

**Figure 6:**
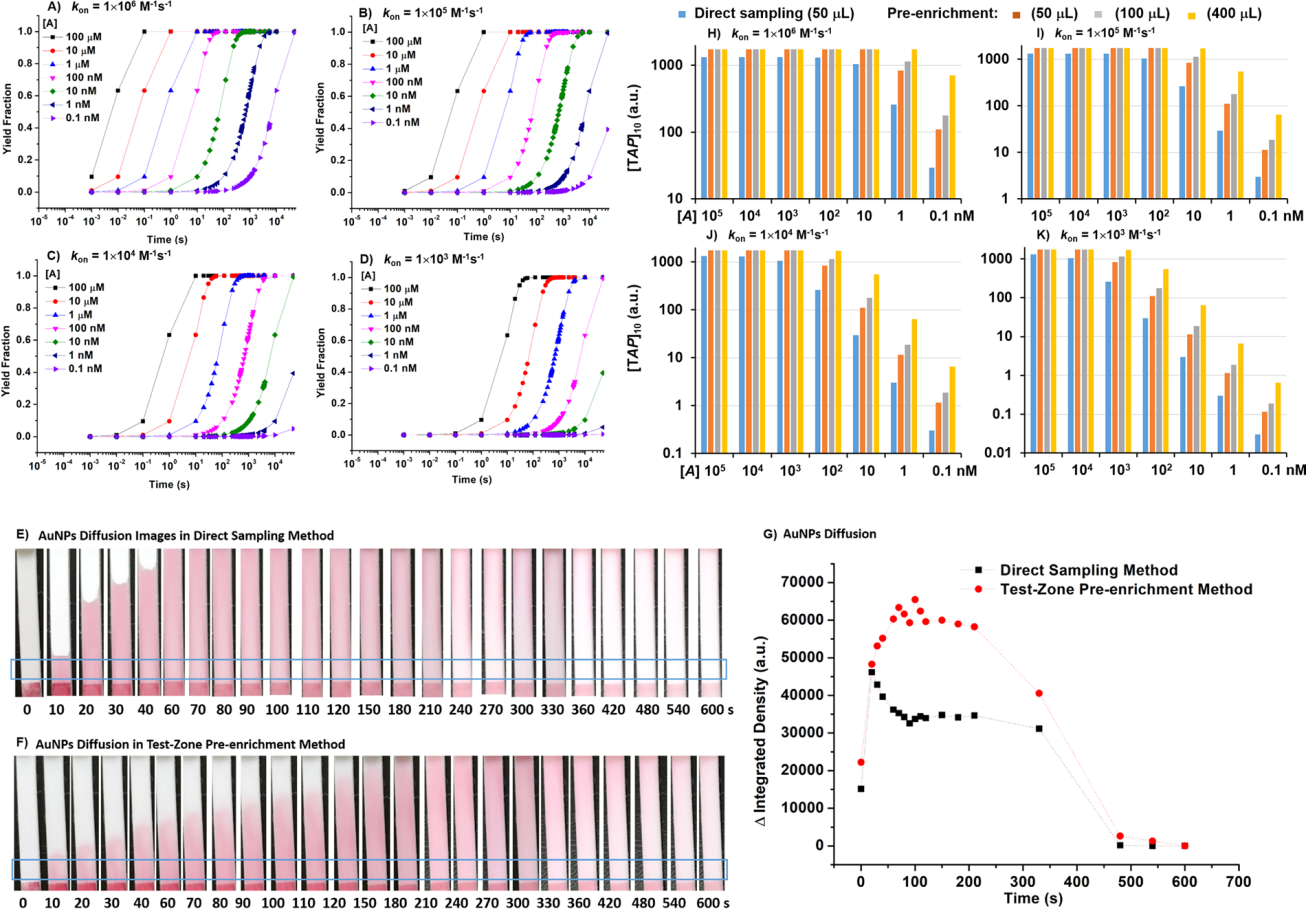
(**A**–**D**) *TA* formation on the test zone of the strip. The moment of sample solution front reaching test zone is set as T = 0 s. (**E**,**F**) AuNPs diffusion images and (**G**) dynamics in the lateral flow assays with direct sampling method and test-zone pre-enrichment method. Only buffer solution was loaded after conjugate pad addition and the volumes of buffer solution are both 50 μL in two methods. The moment of buffer solution front reaching NC membrane is set as T = 0 s after conjugate pad addition, and the LFA is developed about 10 min. The integrated densities of gray in blue box were analyzed by ImageJ to investigate the diffusion of AuNPs. (**H**–**K**) *TAP* formation statistic on the test zone of the strip after DP-AuNPs loading for 10 min.

We used modeling to investigate the impact of sampling method on AuNPs-detecting probe conjugates (DP-AuNPs) capture in molecular-beacon based sandwich-like miR-210 LFA. The model assumptions include: (1) diffusion limits the delivery of DP-AuNPs to the test site, (2) reaction ultimately limits the capture of DP-AuNPs to the test site in the LFA systems, and (3) the reaction of DP-AuNPs capture is kinetically limited second order reversible interactions^17^. As the reaction of DP-AuNPs capture was reported to be the rate-limiting step to improve the LFA sensitivity^10^, we focused on the reaction rate comparison between the direct sampling and the test-zone pre-enrichment method (details in Supplementary Materials). According to the modeling results, the capturing rate of DP-AuNPs (Fig. 6H–K) was higher in the test-zone pre-enrichment method because the concentration of test zone probe-analyte conjugate was much higher (Fig. 6A–D) when DP-AuNPs was loaded (Fig. 6E–G). Given both direct sampling method and test-zone pre-enrichment method were set to read the signal intensity about 30 min after loading DP-AuNPs when the gray intensity of NC membrane recovered to the original value, the capturing rate of DP-AuNPs dominated the amount of captured DP-AuNPs, the decisive factor of sensitivity. Thus, higher sensitivity could be obtained due to the much higher concentration of test zone probe-analyte conjugate in the test-zone pre-enrichment method than that of direct sampling method. This modeling gives us insight into the design and optimization of pre-enrichment enhanced LFAs.

### Selectivity of the test-zone pre-enrichment method

To investigate whether the process of test-zone enrichment will also enhance the analogs’ response, the selectivity of the developed test-zone pre-enrichment method for miR-210 versus its mutants was tested by comparing with that of direct sampling method. LFAs of competitive format I were applied and a variety of miR-210’s mutants with one or three bases mismatched were evaluated. There was no obvious difference in selectivity between direct sampling and pre-enrichment sampling 50 μL of sample solutions (Fig. S12 and Table S3). Impressively, with the increase of enriching volume to 400 μL, except for 5'−1mm, the selectivity was improved with the test-zone pre-enrichment. The possible reason is still under researched.

### Analysis of mock clinical samples

We evaluated the capability of the developed method to improve the sensitivity of miR-210 in spiked serum samples. Pre-enriching 50 μL of pure serum samples gave a LOD of 0.5 pmol in the sandwich-like format (Fig. S13 and Table 1). However, there was not any signal in either test zone or control zone when 50 μL of pure serum samples with miR-210 mimic (up to 50 pmol) was loaded with direct sampling method, possibly because the binding ability between nucleotides is poor in pure serum on the condition that the sample pad has not been pre-treated by buffer solution before usage^31^. In both sandwich-like and competitive formats, LODs obtained by pre-enrichment at 1:1 dilution were equivalent to those obtained in running buffers, indicating that the proposed test-zone pre-enrichment sampling strategy had promising analytical applications in real biological samples. Although the target were detected in properly diluted serum samples in both formats with the direct sampling method, the LODs (50 pmol) achieved in the buffer-diluted serum samples were at least one order of magnitude higher than those (5 nM) obtained in pure buffer solutions. Thus, signals were enhanced and improved, and lower LODs were achieved with pre-enrichment sampling method applied than with direct sampling method in both formats.

**Table 1:** Visual LOD of miR-210 mimic spiked in human blood serum samples.

sampling method	visual LOD (pmol)					
	direct sampling (50 μL)			pre-enrichment (50 μL)		
v/v (serum/buffer)	1:0	1:1	1:3	1:0	1:1	1:3
sandwich-like format	—^a^	—	50	0.5	0.05	0.05
competitive format I	—	50	50	5	5	5

a: no detectable signal by eye.

## Materials and methods

### Chemicals and materials

All the chemicals used are of AR level unless otherwise stated and used as received without purification. Gold (III) chloride hydrate was from Sigma Aldrich (St Louis, MO, USA.). BSA (BR), trisodium citrate, sodium acetate, sodium dodecyl sulphate (SDS, CP), tris(hydroxymethyl)-methyl-aminomethane (Tris), Tween-20 (CP), Na_3_PO_4_·12H_2_O, Na_2_HPO_4_·12H_2_O, KH_2_PO_4_, KCl, NaCl, MgCl_2_, sucrose, glacial acetic acid, NaOH were from Sinopharm Chemical Reagent (Beijing, China). PBS consisted of 1.47 mM KH_2_PO_4_, 8.1 mM Na_2_HPO_4_, 137.92 mM NaCl and 2.67 mM KCl.

Ultrapure water from Wahaha group Co. (Hangzhou, China) was used throughout the experiment. All the oligonucleotide probes and targets were procured from Sangon Biotech Co., Ltd (Shanghai, China). The sequences of nucleotides are shown in Table S1. HCG protein, paper materials including Sartorius CN95 NC membrane, H5072 cellulose pad as absorbent pad, GL-b06 glass fiber as sample pad and DB-8 backing card were from Shanghai JieYi Biotech. Co., Ltd (Shanghai, China). Commercial HCG strips of David brand manufactured by Runbio Biotech Co. Ltd were purchased from a local drug store. AFB_1_ (4-methoxy-2,3,6*α*,9*α*-tetrahydrocyclopenta[*c*]furo[3′,2′:4,5]fu-ro[2,3-*h*]chromene-1,11-dione; certified reference material) was purchased from Beijing Century Aoke Biotech. Co., Ltd (Beijing, China). Test strips for AFB_1_ were from Jiangsu Meizheng Biotech. Co., Ltd (Wuxi, China).

The synthesis and characterization of gold nanoparticles (AuNPs) according to published procedures^32,33^ with some modifications, and preparation of AuNPs-detecting probe conjugates for miR-210 mimic detection were in the Supplementary File.

### Strip assembly and LFA procedures

The assembly of conventional LFA test strips for miR-210 mimic was performed manually according to Storhoff *et al*.^32^ while no conjugating pad was assembled into the test strips before sampling in pre-enrichment method. After assembly, membranes were cut into 4 mm width strips. 0.5 μL of 100 μM test zone probe and control zone probe in ultrapure water were dispensed as a test and control, respectively. Then, the strips were dried at 65 °C for 30 min and stored in valve bags at room temperature. We constructed LFAs for miR-210 mimic in both sandwich-like and competitive formats and the sequences of nucleotides used here are shown in Table S1. The procedures for direct sampling and pre-mixing sampling method were carried out according to Storhoff *et al*.^32^ while test-zone pre-enrichment method proceeded as follows. Briefly, the strips comprising the wicking pad, the NC membrane and the sample pad (without the conjugate pad) were inserted into the tubes of sample in running buffer (PBS, pH 7.4, containing 4% BSA and 1% SDS) with the sample pad submerged in the liquid, and the fluid wicked through the strips towards the absorbance pad to carry out pre-enrichment and separation. After 8-min pre-enrichment, a piece of conjugate pad (6.5 mm × 4 mm) containing dry AuNPs-detecting probe conjugates (DP-AuNPs) was put on the sample pad and 20 μL of running buffer was added onto the conjugate pad. Ten minutes later, the signals on the strips were stable and we got visual result.

To get the results of semi-quantitative analysis, the strips were dried at 30 °C for 10 min, scanned with HP LaserJet Pro M1136 scanner and analyzed by ImageJ software for quantification of the relative integrated density of gray (T/C). All assays were performed three times at room temperature. In competitive formats, the lower initial point of linear range was taken to calculate the semi-quantitative LOD.

Except for miR-210, we also examined HCG and AFB_1_ as model targets of protein molecule and small organic molecule, respectively. Sample solutions were directly loaded onto commercial test strips in direct sampling method, while the conjugate pads were taken out before sample loading and put back onto sample pads after sample loading in test-zone pre-enrichment method.

A small-scale validation study was conducted at Jiangnan University after obtaining approval from the Jiangnan Institutional Review Board. Serum samples were from volunteers recruited via an advertisement during health check. A trained nurse collected venipuncture blood samples from each volunteer. The venipuncture blood samples were centrifuged and original or diluted serum samples were immediately dispensed onto the inlet of the LFA test strip for the miR-210 test as described in the protocol above or stored in a − 20 °C refrigerator until analysis. All experiments were performed in compliance with the relevant laws and institutional guidelines. All experiments were approved by Jiangnan Institutional Review Board. And we have obtained the informed consent of all volunteers.

## Supplementary information

Supplementary Information.
