# Supplementary material for: Improvement in Detection Limit for Lateral Flow Assay of Biomacromolecules by Test-Zone Pre-enrichment
Source: Sci Rep. 2020 Jun 15;10:9604. doi: 10.1038/s41598-020-66456-1 (PMC7295814; doi:10.1038/s41598-020-66456-1)
Supplement: Supplementary file 1 — Supplementary Information. [file 41598_2020_66456_MOESM1_ESM.pdf]

Supplementary Material

Improvement in Detection Limit for Lateral Flow Assay of Biomacromolecules by Test-Zone Pre-enrichment

Yi Zhang [1,*], Xiao Liu [1], Lingling Wang [1], Hanjie Yang [1], Xiaoxiao Zhang [1], Chenglong Zhu [1], Wenlong Wang [1], Lijing Yan [2], Bowei Li [3]

[1] State Key Laboratory of Food Science and Technology, International Joint Laboratory on Food Safety, Collaborative innovation center of food safety and quality control in Jiangsu Province, Institute of Analytical Food Safety, School of Food Science and Technology, Jiangnan University, Wuxi, 214122, PR China

[2] Jiangnan University Hospital, Wuxi, 214122, PR China

[3] CAS Key Laboratory of Coastal Environmental Processes and Ecological Remediation, Research Center for Coastal Environmental Engineering and Technology, Yantai Institute of Coastal Zone Research, Chinese Academy of Sciences, Yantai, 264003, PR China

***Corresponding author**
E-mail: zhangyijnu@jiangnan.edu.cn

Synthesis of gold nanoparticles

Gold nanoparticles (AuNPs) for miR-210 mimic detection were synthesized according to published procedures [1,2] with some modifications. After the color turned from pale yellow into deep red with the addition of trisodium citrate solution, the hot stirring-heater was exchanged with a room-temperature stirrer as soon as possible. The synthesized AuNPs were filtered through a 0.45 μm polyether sulphone filter and stored in 4 ^{o}C as AuNPs stock solution. The concentration of gold in AuNPs stock solution was about 0.9 mM according to the amount of raw materials.

Preparation of AuNPs-detecting probe conjugates and the conjugate pad

Conjugation of detecting probe (DP), which is oligonucleotide molecule here, with gold nanoparticles was performed in 10 mL plastic centrifugation tubes. To optimize the conjugating ratio of thiolated detecting probe to AuNPs, 0.4 M NaCl solution was added to their mixture of different ratios to make a final concentration of NaCl as high as 0.2 M, and the color change was observed. After 24 h of storage, the proportion in which solution color remained red was chosen. In a typical synthesis, 50 μL of 100 μM thiolated DNA was mixed with 1 mL of AuNPs stock solution. After overnight incubation, 1 mL of 10 mM Tris-acetate buffer of pH 8.2 and 2 mL of 0.4 M NaCl were added to the mixture and incubated for 24 h in dark. The aged conjugates were centrifuged at ~18,000 g for 20 min at 4 ^{o}C and washed with ultrapure water three times. The precipitate was dissolved in 500 μL buffer containing 20 mM Na_3PO_4, 5% BSA, 0.25% tween-20 and 10% sucrose and stored as detecting probe conjugated AuNPs (DP-AuNPs) stock solution.

To make a conjugate pad with DP-AuNPs, 250 μL of DP-AuNPs stock solution was sprayed onto a conjugate pad (0.65 cm × 30 cm) using a 3D spray point platform, dried in a vacuum at room temperature, stored in a dryer at 4 ^{o}C and cut into 6.5 mm × 4 mm.

Characterization of DP-AuNPs

The effective combination of AuNPs and DP molecules is one of the key factors for the success of LFA. The optimal ratio of DP to AuNPs for the preparation of DP-AuNPs was studied in presence of 0.2 M NaCl, with an ionic strength level high enough to precipitate AuNPs that were not fully protected (**Fig. S1**). Upon addition of NaCl, solutions with molar ratios lower than 10 pmol DP to 9.1 nmol Au showed color change from wine red to blue immediately, which is the typical phenomenon of AuNPs aggregation, while those with higher molar ratios tolerated the presence of 0.2 M NaCl well. The produced solution with a molar ratio

of 10 pmol DP to 9.1 nmol Au turned to bluish violet after 24 h of storage/aging, indicating this molar ratio was still not high enough to prohibit the self-assembling of AuNPs into bigger aggregates induced by high concentration of salt in long-term storage, while molar ratios of 50 pmol DP to 9.1 nmol Au seemed high enough. Then the performance of as-synthesized DP-AuNPs in sandwich-like LFA was verified. From the inset of Figure S1, DP-AuNPs synthesized with higher molar ratio of DP/AuNPs showed better signal at both control and test zone with the presence of target oligonucleotide at the same concentration. So the molar ratio of 100 pmol DP to 9.1 nmol Au was chosen for further synthesis [1]. The AuNPs and DP-AuNPs were characterized by UV-vis absorption spectrometry, and transmission electron microscopy (TEM) (**Fig. S2**). Both AuNPs and DP-AuNPs were about 20-30 nm in diameter according to their TEM image and their maximal absorption at 521.5 nm for AuNPs and 526.5 nm for DP-AuNPs. The UV-vis spectrum of DP-AuNPs showed slight red shift as compared with that of AuNPs, verifying the successful fabrication of DP-AuNPs conjugates.

Simulation and modeling

The model assumptions include: (1) diffusion limits the delivery of DP-AuNPs to the test site, (2) reaction ultimately limits the capture of DP-AuNPs to the test site in the LFA systems, and (3) the reaction of DP-AuNPs capture is kinetically limited second order reversible interactions.

As the reaction of DP-AuNPs capture was reported to be the rate-limiting step to improve the LFA sensitivity, we focused on the reaction rate comparison between the direct sampling and the test-zone pre-enrichment method.

In molecular beacon sandwich-like format we used, only when the analyte bound with the ring part of the molecular beacon (MB, as capture probe) at test zone probe and disassembled the self-binding at the stem of MB, the detecting probe on AuNPs could bind with the 3' end of MB and showed a visible signal at test zone. Thus there are two successive essential reactions for the capture of AuNPs on the test zone,

1) $T + A \rightleftharpoons TA$

2) $TA + P \rightleftharpoons TAP$

In reaction 1, two species including analyte (*A*) and test zone probe (*T*) are involved, and the product the conjugate of test zone probe and analyte (*TA*) is one reactor of reaction 2. The formation of *TAP* (conjugate of *TA* and *P* (DP-AuNPs) ultimately limits the capture of DP-AuNPs to the test zone and is the decisive factor of final color intensity.

i) Analyte capture

The concentration of *TA* (conjugate of test zone probe and analyte) can be solved from [3-5],

$$\frac{d[TA]}{dt} = k_{on} \times ([T]_0 - [TA]) \times [A] - k_{off} \times [TA] \quad \text{(Eq. 1)}$$

k_{on} and k_{off} are forward and backward reaction rate constants, $[T]_0$ is the initial concentration of test zone probe. The concentration of analyte *A* is considered constant as $[A]_0$.

Analytical solution for the concentration of *TA* is,

$$[TA] = \frac{k_{on} \times [T]_0 \times [A]_0}{k_{on} \times [A]_0 + k_{off}} (1 - e^{-(k_{on} \times [A]_0 + k_{off})t}) \quad \text{(Eq. 2)}$$

Therefore, *TA* increases exponentially with the increase of reaction time *t*. k_{off} could be ignored when $k_{on} \times [A]_0$ is larger than $20 \times k_{off}$ here. Then, analytical solution for the concentration of *TA* could be,

$$[TA] \approx [T]_0 (1 - e^{-(k_{on} \times [A]_0)t}) \quad \text{(Eq. 3)}$$

$[T]_0$ is constant and is assumed to be 100 μM. The forward reaction rate constants (k_{on}) for the reaction 1 are predicted to be 1.28-2.70×10^6 $M^{-1}s^{-1}$ with salinity of 0.01-1 via a web-based software tool available at http://nablab.rice.edu/nabtools/kine constructed by Zhang et al. in 2018 [6]. There must be some deviation from the reality because the hybridization experimentally characterized in their work were all performed in 5×PBS buffer solution but the reactions takes place on NC membrane here, and all their target and probe sequences were 36 nt long but ours are only 24 nt long. [*TA*] versus reaction time curves are simulated by assuming hybridization rate constants to be 1×10^6 $M^{-1}s^{-1}$ to 10^3 $M^{-1}s^{-1}$ as almost all the k_{on} in ref 6 (k_{Hyb} was used in the literature) were within that range (**Fig. 6a to 6d)**.

As the former one of a two-step continuous reaction, the more fully balanced reaction 1 will benefit the subsequent reaction a lot. Therefore, when the reaction rate constant is fixed and the concentration of the target is constant, appropriate extension of the reaction time of the analyte on the test strip is conducive to the more sufficient reaction 1, so that more analyte molecules can be captured in the test zone. Usually, the LFA takes 10-30 minutes to complete the test. If the combined reaction can reach a balance within this period, the best detection effect may be achieved. As for different $[A]_0$ (100 μM, 10 μM, 1 μM, 100 nM, 10 nM, 1 nM and 0.1 nM) at certain assumed *T* and k_{on}, the higher $[A]_0$ is, the faster *TA* forms, and vice versa (**Fig. 6a**). Given k_{on} is 1×10^6 $M^{-1}s^{-1}$, it takes less than 0.1 s for the reaction of *TA* formation to reach balance

if $[A]_0$ is 100 μM, 1 s for 10 μM $[A]_0$, 500 s for 10 nM $[A]_0$, while up to 2.5 h is needed if $[A]_0$ is only 1 nM. Thus, when the target concentration is high, the reaction 1 can quickly reach balance, and the subsequent signaling unit's capture becomes the speed limit step. However, when the concentration of the target is low, the reaction 1 can hardly reach balance within one test duration, then the reaction 1 turns to be the speed limit step. If the sample solution is pre-loaded for enrichment for 10-20 minutes, the analyte with the concentration at 10 nM or above will be able to fully react with the capture probe and reach the reaction balance. The enrichment of analyte and the increase of *TA* products in the test zone undoubtedly provide a great promotion to the follow-up reaction 2 in the test zone.

The time difference of the binding reaction with different reaction rate constants to reach equilibrium on the test strip will also affect the result of the LFA. While $[A]_0$ is fixed as 100 μM, it takes only 0.1 s for the reaction of *TA* formation to reach balance if k_{on} is 1×10^6 $M^{-1}s^{-1}$, however up to 100 s is needed if k_{on} is only 1×10^3 $M^{-1}s^{-1}$ (**Fig. 6d**). Therefore, in order to realize the rapid detection of test strip, it is necessary to select antibodies or adapters with a binding rate constant high enough to facilitate the rapid *TA* and *TAP* formation in a limited time.

ii) AuNPs diffusion/[P] profile

There are two consecutive phases of flow in the LFAs: "membrane" and "absorbent pad" phase, and the transition point from "membrane flow" to "absorbent pad flow" is the moment that the solution frontier reaches the absorbent pad. Time dependent velocity ($U = a \times t^{-\frac{1}{2}}$, *U* is the diffusion velocity, *a* is a constant related with the pore diameter of NC membrane, t is the diffusion time) could be obtained from "membrane flow", while the slower velocity from "absorbent pad flow" is constant. Exactly, with the direct sampling method, DP-AuNPs travels through NC membrane in "membrane flow" within the first minute and in "absorbent pad flow" in the following minutes **(Fig. 6e)**. However, DP-AuNPs travels only in "absorbent pad flow" with the test-zone pre-enrichment method because the NC membrane is already thoroughly wetted after sample pre-enrichment **(Fig. 6f)**. We calculated the diffusion of AuNPs according to the integrated density of gray of the squared part assuming the velocity of AuNPs here is the same as that at the test zone and achieved the dynamics of AuNPs in different sampling method **(Fig. 6g)**. In the direct sampling method, the concentration of AuNPs in the front end of the solution was the highest, and it fell to less than 82% within 50 s. However, in the pre-enrichment method, the concentration of AuNPs peaked and fell more slowly, and it maintained at more than 87% for at least 210 s. In addition, the peak concentration of AuNPs

in the pre-enrichment method seemed to be larger than that achieved in different sampling method although AuNPs were loaded equally in the two methods. Therefore, with the reduced flow rate and longer duration of high AuNPs concentration, the proposed test-zone pre-enrichment could introduce increased AuNPs capture interaction at test-zone.

iii) [*TAP*] profile

According to reaction 2, the concentration of *TAP* can be solved from,

$$\frac{d[TAP]}{dt} = k_{on}' \times [P] \times [TA] - k_{off} \times [TAP] \quad \text{(Eq. 4)}$$

$k_{off} \times [TAP]$ could be ignored because k_{off} and [*TAP*] are both several orders of magnitude smaller than k_{on} here. Thus Eq. 4 could be simplified to,

$$\frac{d[TAP]}{dt} \approx k_{on}' \times [P] \times [TA] \quad \text{(Eq. 5)}$$

With given sampling method, k_{on}' is [*A*]-independent. According to [*P*] profile, [*P*] is also [*A*]-independent. Therefore, [*TAP*] is [*A*]- and k_{on}-dependent via [*TA*].

For different [*A*] (100 μM, 10 μM, 1 μM, 100 nM, 10 nM, 1 nM and 0.1 nM), [*TAP*]-time curves are simulated **(Fig. S8)**. Under the assumed reaction constant k_{on} ranging from 1×10^{6} $M^{-1}s^{-1}$ to 1×10^{3} $M^{-1}s^{-1}$, the final [*TAP*] and the slopes of [*TAP*]-time curves for direct sampling method are all smaller than that for test-zone pre-enrichment method.

In direct sampling method, DP-AuNPs migrates along with the analyte toward the test zone and reacts there. When they arrive at the test zone, the reaction to generate *TA* has just started, thus [*TA*] is quite low at the initial stage of LFA **(Fig. 6a)**. Therefore, the reaction rate of *TAP* formation is mainly controlled by [*TA*] and is very slow at the initial minutes.

However, in the test-zone pre-enrichment method, when DP-AuNPs is loaded a large amount of *TA* has already been generated in the test zone after minutes of pre-enrichment of analyte under the same concentration of analyte as that of the direct sampling method. Therefore, the rate of *TAP* formation is greatly increased with the larger [*TA*] and the final [*TAP*] is also improved in the test-zone pre-enrichment method compared with that of direct sampling method.

According to the simulation, $[TAP]_{10}$ ($[TAP]$ achieved 10 min after loading of DP-AuNPs) versus $[A]$ are analyzed **(Fig. 6h to 6k)**. In the direct sampling method (group 1), given k_{on} is 10^6 or 10^5 $M^{-1}s^{-1}$, there is no difference in $[TAP]$ when $[A]$ is 1 μM or 10 μM, while given k_{on} is 10^3 $M^{-1}s^{-1}$, the target seems undetectable even at 10 nM sampling 50 or 100 μL with pre-enrichment method, which are both not consistent with the experimental results in Figure 2a. Only the statistic results with the assumption of k_{on} as 10^4 $M^{-1}s^{-1}$ seem to be consistent with most of the experimental results: 1) there is a linear relationship between the signal at test zone and log$[A]$ from 10^5 nM to 10 nM in direct sampling method and from 10^3 nM to 1 nM in pre-enrichment method; 2) the signal of 50, 100 and 400 μL analyte at 10 nM and 400 μL analyte at 1 nM in pre-enrichment method is comparable with that of 50 μL analyte at 100 nM in direct sampling method and larger than that of 50 μL analyte at 10 nM in direct sampling method, which is undetectable in experiment . Therefore, we may say that k_{on} is possibly 10^4 $M^{-1}s^{-1}$ for reaction 1), and with the pre-enrichment of 50, 100 and 400 μL 10 nM sample solution, $[TAP]_{10}$ could be increased by 3.7-fold, 6.0-fold and 18.6-fold compared with that of direct sampling method, leading to improved LOD. $[TAP]_{10}$ is still detectable with pre-enrichment 400 μL 1 nM sample solution. Given both methods were set to read the signal intensity 10 min after loading DP-AuNPs, the test-zone pre-enrichment method was able to generate more *TAP* to obtain higher sensitivity due to the much higher initial $[TA]$.

According to the modeling results, the capturing rate of DP-AuNPs was higher in the test-zone pre-enrichment method because the concentration of test zone probe-analyte conjugate was much higher when DP-AuNPs was loaded. Given both direct sampling method and test-zone pre-enrichment method were set to read the signal intensity 10 min after loading DP-AuNPs when the gray intensity of NC membrane recovered to the original value (**Fig. S9 to S11**), the capturing rate of DP-AuNPs dominated the amount of DP-AuNPs captured, the decisive factor of sensitivity. Thus, higher sensitivity could be obtained due to the much higher concentration of test zone probe-analyte conjugate in the test-zone pre-enrichment method than that of direct sampling method. This modeling gives us insight into the design and optimization of pre-enrichment enhanced LFAs.

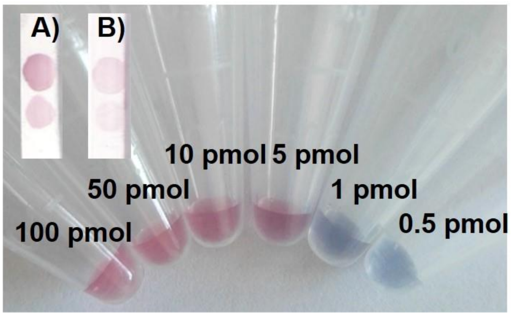

Fig. S1 Color images of the mixture of 10 μL AuNPs stock solution (with 9.1 nmol Au element) and different amounts of detecting DNA probe at 0.2 M NaCl. The products at 100 pmol and 50 pmol detecting DNA probe were chosen to proceed LFA at exactly same conditions and the results were shown in inset (A) and (B)

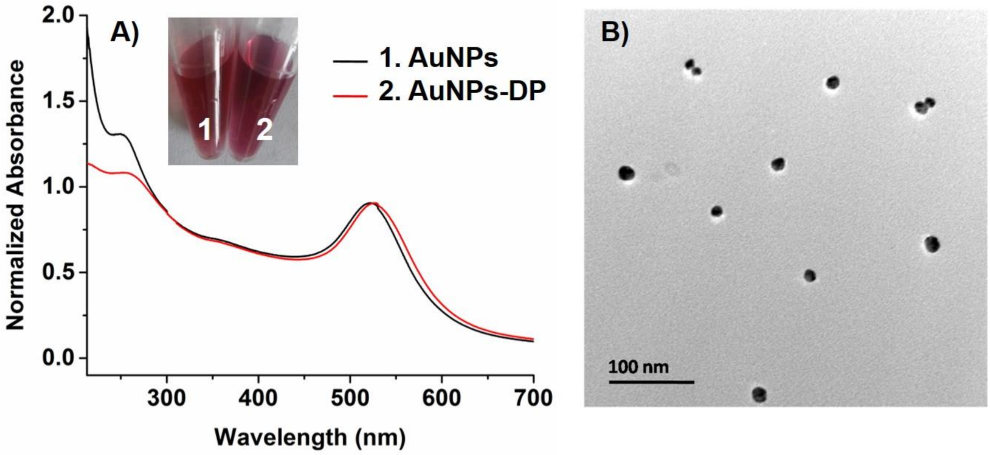

Fig. S2 (A) UV/Vis absorption spectra of AuNPs (1) and AuNPs-DP (2) and (B) TEM photo of AuNPs-DP

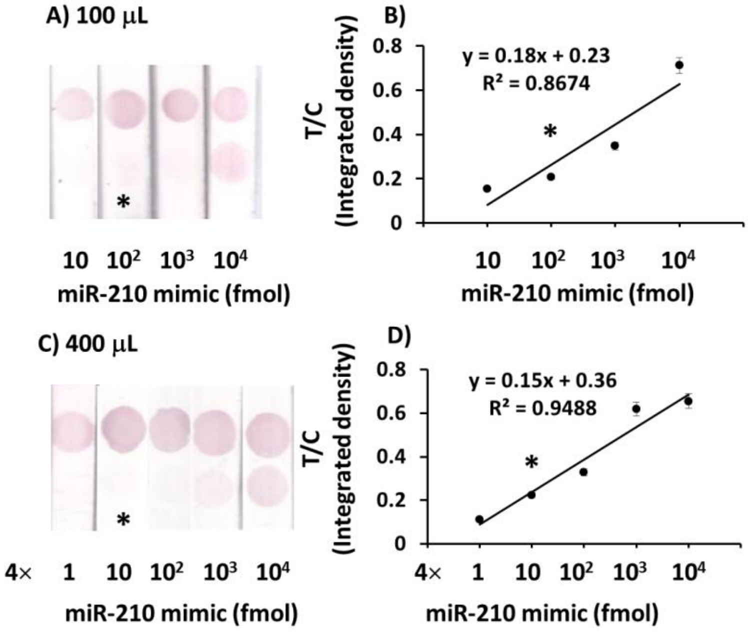

Fig. S3 Test-zone pre-enrichment of sandwich-like format

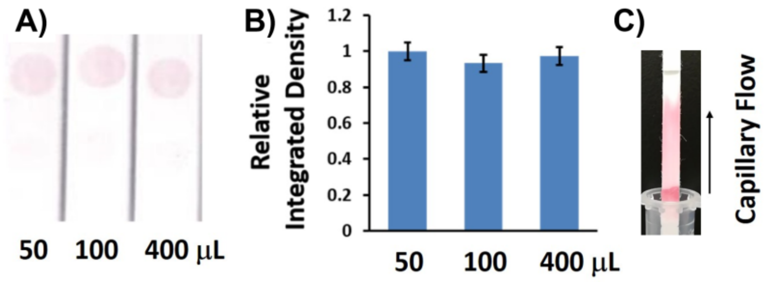

Fig. S4 Direct sampling different volumes of 10 nM miR-210 mimic by sandwich-like format. (A) Images of LFA strips. (B) Analysis results by ImageJ. (C) One real-time image of the separation in LFA

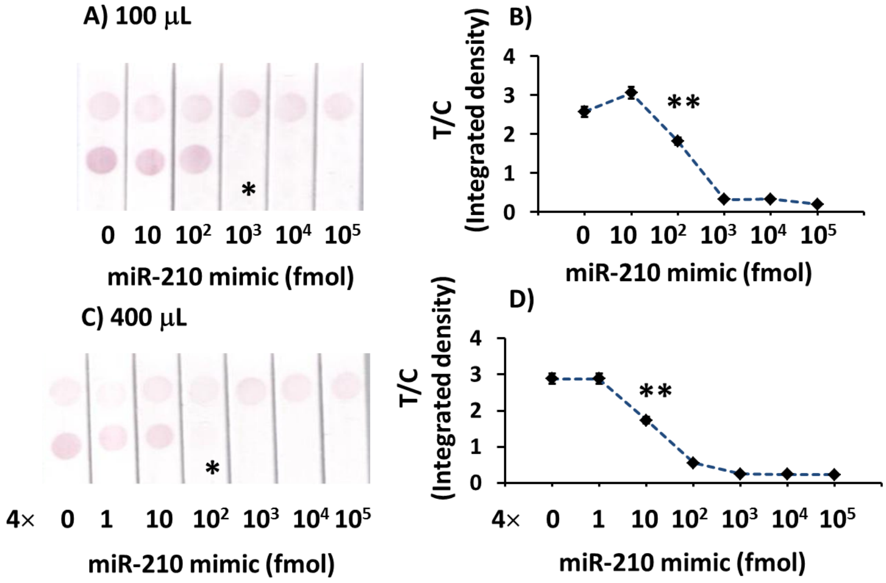

Fig. S5 Test-zone pre-enrichment results of competitive format I LFAs for miR-210. Single asterisk (*) indicates the cutoff value as visual LODs, while double asterisk (**) indicates the cutoff value as semi-quantified LODs

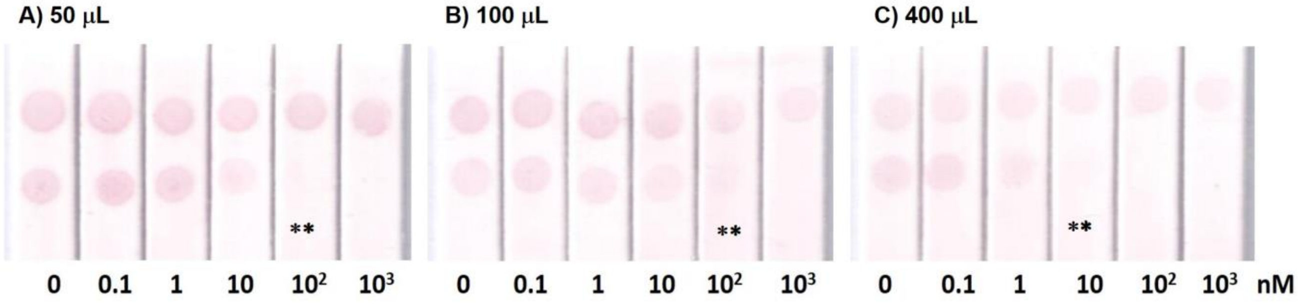

Fig. S6 LFA images of competitive format I got by pre-mixed signal unit with (A) 50 µL, (B) 100 µL and (C) 400 µL target (miR-210 mimic) at different concentrations, respectively. Double asterisks (**) indicate cutoff value as visual LODs

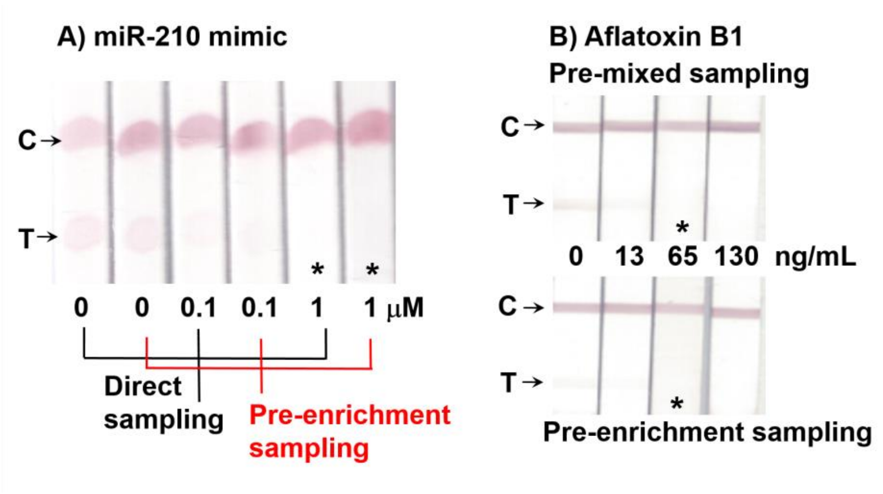

Fig. S7 Images of competitive format II LFAs for (A) miR-210 with direct sampling and test-zone pre-enrichment sampling methods and (B) aflatoxin B1 with pre-mixed sampling and test-zone pre-enrichment sampling methods. Single asterisk (*) indicates the cutoff value as visual LODs

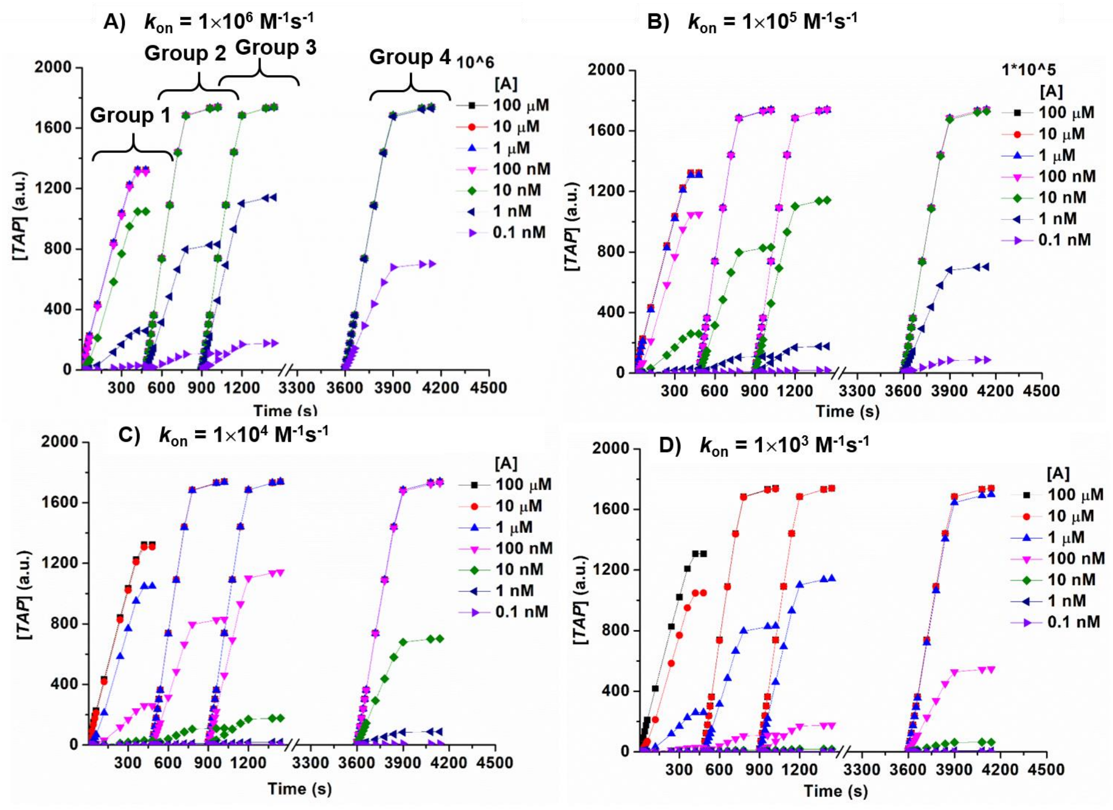

Fig. S8 *TAP* formation dynamics on the test zone of the strip. The moment of sample solution front reaching test zone is set as T = 0 s. Group 1: direct method, 50 μL; group 2: pre-enrichment, 50 μL; group 3: pre-enrichment, 100 μL; group 4: pre-enrichment, 400 μL

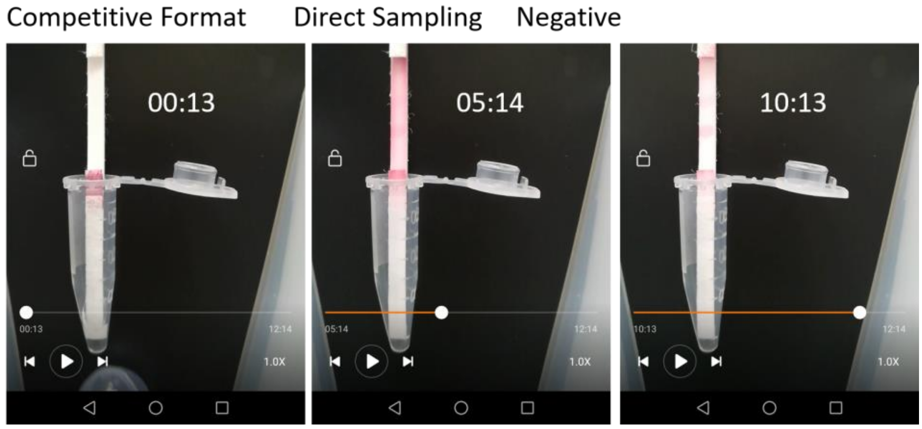

Fig. S9 Dynamic images of competitive format I LFAs by direct sampling

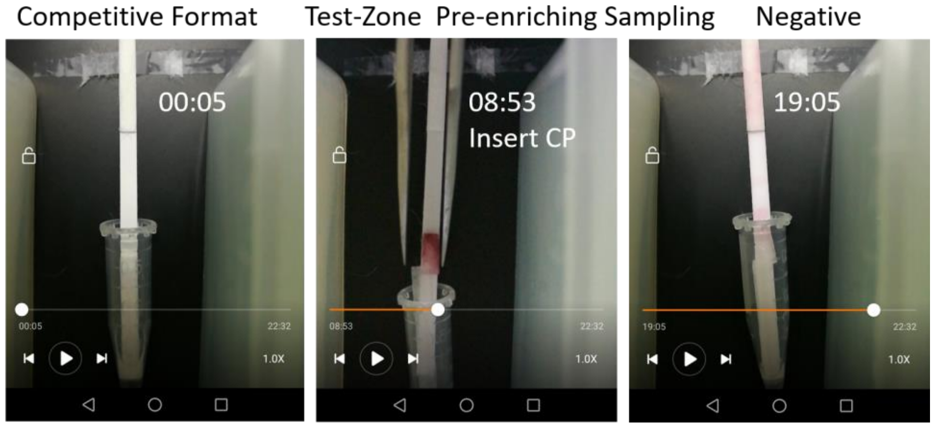

Fig. S10 Dynamic images of competitive format I LFAs by test-zone pre-enrichment method

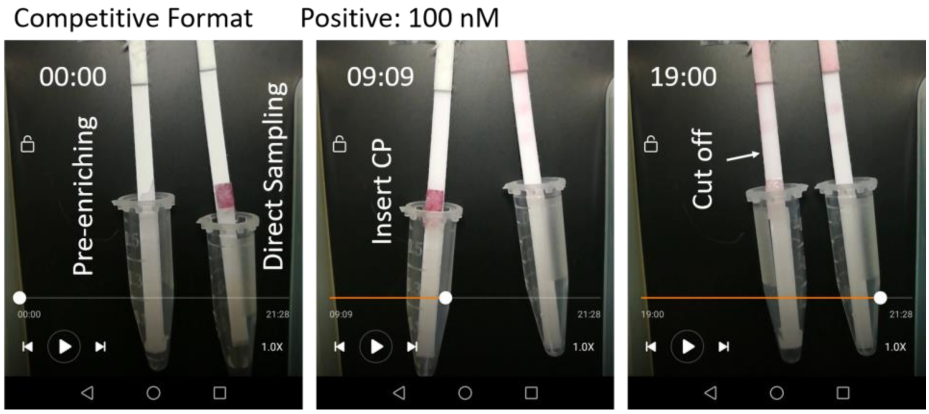

Fig. S11 Dynamic images of competitive format I LFAs by direct sampling and test-zone pre-enrichment method

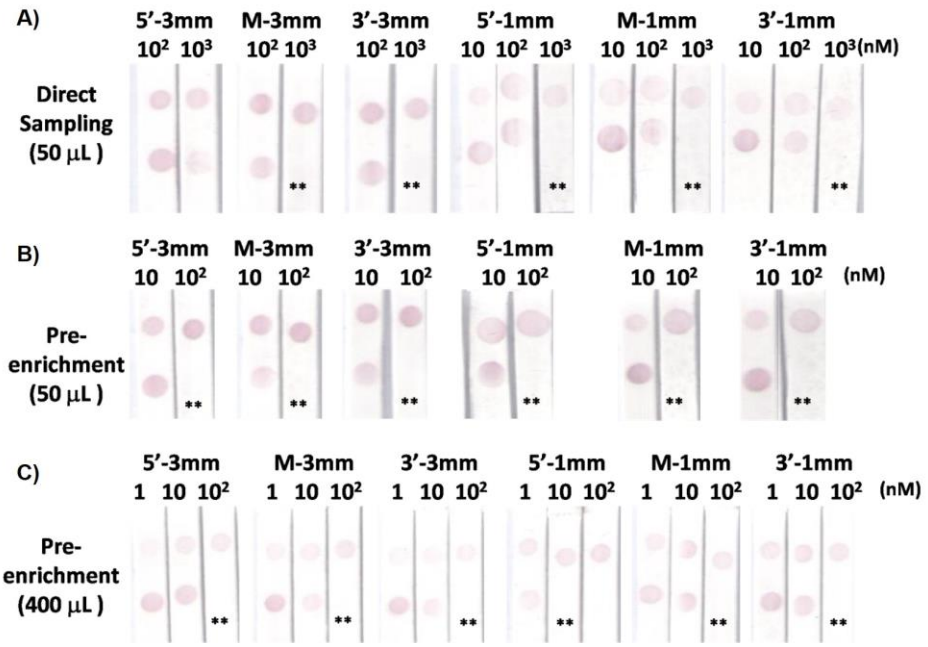

Fig. S12 LFAs of mismatch variants of miR-210 mimic by competitive format. Double asterisks (**) indicate cutoff value

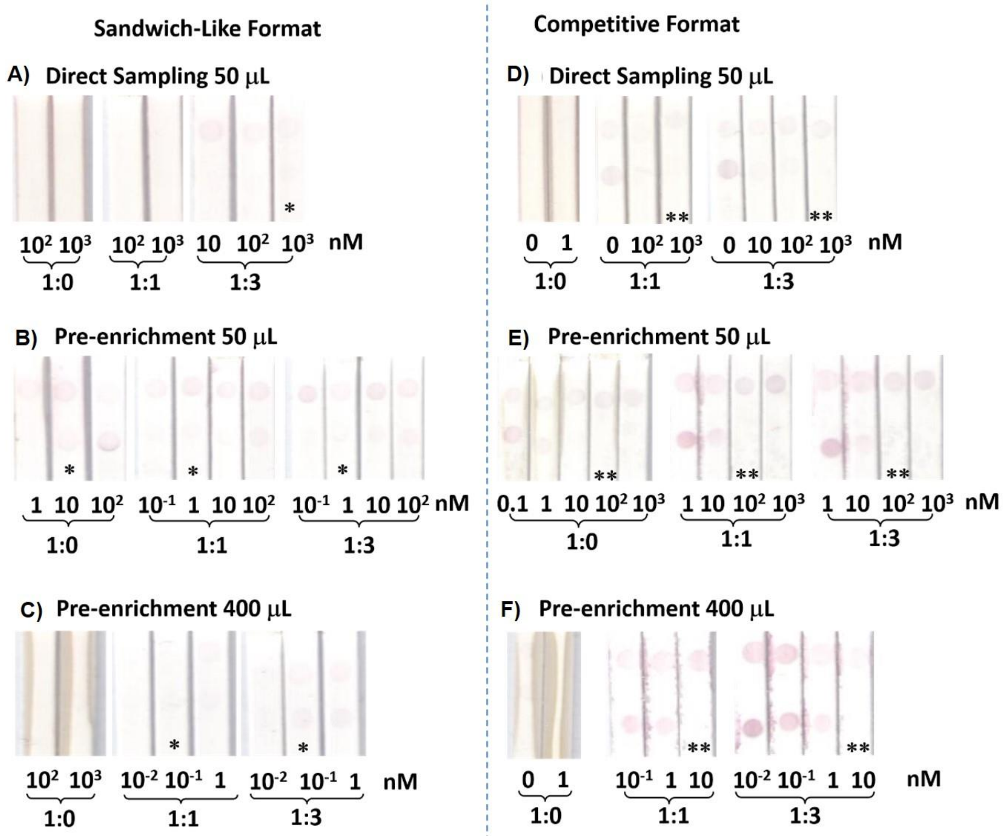

Fig. S13 LFAs of miR-210 mimic at different concentrations spiked in the human blood serum in sandwich-like and competitive format. 1:0, 1:1 and 1:3 are the volume ratio of the serum and the running buffer. Single asterisk (*) indicates the visual LOD in sandwich-like format, while double asterisks (**) indicate the cutoff values as visual LOD in competitive format.

Table S1

The DNA oligonucleotide sequences for LFA

	name	sequence (5’ to 3')
target	miR-210 mimic	CTGTGCGTGTGACAGCGGCTGA
mismatch- variants	5'-1mm[a]	ATGTGCGTGTGACAGCGGCTGA
	5'-3mm	AGTTGCGTGTGACAGCGGCTGA
	M[b]-1mm	CTGTGCGTGTTACAGCGGCTGA
	M-3mm	CTGTGCGTGGTCCAGCGGCTGA
	3'-1mm	CTGTGCGTGTGACAGCGGCTGC
	3'-3mm	CTGTGCGTGTGACAGCGGCGTC
sandwich-	detecting probe	HS-$(CH_2)_6$-A10GTAAAACGACGGCCAGT
like format	test zone probe	A15GTAAAACGACGGCCATCAGCCGCTGTCACACGCACAGACTGGCCGTCGTTTTAC
	control zone probe	A40ACTGGCCGTCGTTTTAC
competitive format I	detecting probe	HS-$(CH_2)_6$-A10CTGTGCGTGTGACAGCGGCTGA GTAAAACGACGGCCAGT
	test zone probe	A15TCAGCCGCTGTCACACGCACAG
	control zone probe	A40ACTGGCCGTCGTTTTAC
	detecting probe	HS-$(CH_2)_6$-A10TCAGCCGCTGTCACACGCACAG GTAAAACGACGGCCAGT
competitive format II	test zone probe	A15CTGTGCGTGTGACAGCGGCTGA
	control zone probe	A40ACTGGCCGTCGTTTTAC

[a] mm: mismatch; [b] M: middle; nucleotides with underline are mismatched sites

Table S2

Comparison of the analysis time with other reported lateral flow assays

ref	target	time (min)	improvement	notes
Analyst 2019, 144(5): 1840–1849	cholera toxin	10		
Biosens. Bioelectron. 2018, 113, 95–100	C-Reactive Protein	10		spatial constrictions of the flow-path
Food Chem. 2019, 279: 246–251	P-35s and T-nos	10		dual super PCR and a lateral flow biosensor
Sensor. Actuat. B-Chem. 2018, 262: 486–492	glutathione	10		
Phys. Status Solidi A 2012, 209(5): 917–924	horseradish peroxidase	12		enrichment module based on polyethylene sintered bodies
Anal. Chem. 2017, 89, 12137−12144	PCR products	10-15		graphene oxide purification
Analyst 2018, 143: 4646−4654	morphine and methamphetamine	15		
Anal. Chim. Acta 2018, 1009: 81−88	viral nucleic acids	15		electrospin-coating of nitrocellulose membrane
Food Control 2019, 95: 34−40	microcystin-LR	15		
Nanomed.-Nanotechnol. 2018, 14: 1257−1266	acute myocardial infarction biomarkers	15		
Sci. Rep.-UK 2017, 7: 1360	HBV nucleic acid	15	10-fold ↓ in LOD	sponge pad
Sensors-Basel 2019, 19(1), 153	Ralstonia solanacearum	15		
Sensor. Actuat. B-Chem. 2018, 270: 72−79	high-risk bacterial pathogens	15		
Sensor. Actuat. B-Chem. 2019, 282: 317−321	*Salmonella serogroups*	15		
Sensor. Actuat. B−Chem. 2019, 283: 222−229	benzothiostrobin residue	15		
Sensor. Actuat. B−Chem. 2019, 285:	troponin	15	10-fold ↓ in LOD	a magnetic field assisted

431−437				preconcentration approach
Sensor. Actuat. B−Chem. 2019, 286: 272−281	pepsinogen I/II	15		
Sensor. Mater. 2015, 27, 549–561	DNA	16	50% ↑ in signal intensity	double run signal unit
Anal. Chem. 2015, 87, 1009−1017	IgG labeled with AF488	17	400-fold (90 s) 160-fold (5 min) ↓ in LOD	7 min for isotachophoresis
Anal. Chim. Acta 2018, 1049: 143−149	clenbuterol and ractopamin	18		
Anal. Chem. 2017, 89, 10216−10223	malaria RDT	20	17.5-fold ↓ in LOD	10 min for enrichment
Anal. Chim. Acta 2019, 1053: 139−147	avian influenza A (H7N9)	20		
Anal. Chim. Acta 2019, 1055: 140−147	zika virus	20		
Food Chem. 2018, 269: 375–379	β-conglutin	20	10^3-fold ↓ in LOD	immunomagnetic enrichment and enzyme signal amplification
Sci. Rep.-UK 2018, 8, 17319	protein A and C-reactive protein	20	5- and 2-fold ↓ in LOD	additional stacking pad
Sensor. Actuat. B−Chem. 2018, 273, 1323–1327	troponin I	20	10-fold ↓ in LOD	water-soluble nanofibers and silver-enhancement reactions
Sensor. Actuat. B−Chem. 2019, 285: 326−332	HPV16 DNA	20		
Nano-Micro Lett. 2018, 10: 24	procalcitonin	23	10-fold ↓ in LOD	silver-enhanced hierarchical nanogold
Front. Cell. Infect. Mi. 2019, 9: 1	Trichinella Spp	10-25		recombinase polymerase amplification assay
PLoS One 2015,10, e0142654	hCG	15-25	100-fold ↓ in LOD	aqueous two-phase systems
Talanta 2016, 152, 269–276	HIV nucleicacid, myoglobin	25	10- and 4-fold ↓ in LOD	paper-based sample concentration

Lab Chip, 2014, 14, 3021-3028	transferrin	25	10-fold ↓ in LOD	aqueous two-phase systems
Anal. Chem. 2016, 88, 10701−10710	β-conglutin	30		combined competitive-amplification
Anal. Chem. 2018, 90, 708−715	*S. typhimurium*	30		the universal blocking linker recombinase polymerase amplification
J Dairy Sci. 2018, 101: 8767−8777	aflatoxin M-1 and Escherichia coli O157:H7	30		
Microchim. Acta 2018,185(9): UNSP 404	tetracycline antibiotics	30		including sample treatment
PLoS One 2018,13(11): e0207811	Rickettsia rickettsii	30		recombinase polymerase assay
J Microbiol. Meth. 2019, 159: 56−61	M. hyopneumoniae	37		recombinase polymerase amplification assays
Parasite. Vector. 2015, 8: 241	worm circulating anodic antigen	75		60 for incubation target with signaling-unit, centrifugal filtration
Food Chem. 2019, 274: 803−807	DNA	105		75 min for PCR
Anal. Chim. Acta 2015, 861, 62–68	*Escherichia coli* O157:H7	120		90 min for sample preparation, 30 min for amplification
Sci. Rep.-UK 2019, 9: 393	microbial source tracking marker	120		isothermal helicase-dependent amplification
J Dairy Sci. 2016, 99(12): 11780	*Escherichia coli* O157:H7	300	25-fold ↓ in LOD (7 h)	5 h for sample enrichment
This work	miR-210 mimic, hCG	20 (50 μL)	10- to 100-fold ↓ in LOD	8 min test-zone enrichment (50 μL)

Table S3

The cutoff value (nM) of miR-210 mimic and its mismatch variants in competitive format I

	cutoff value (pmol)						
sampling method	miR-210 mimic	5'-3mm	M-3mm	3'-3mm	5'-1mm	M-1mm	3'-1mm
direct sampling (50 μL)	50	> 50	50	50	50	50	50
pre-enrichment (50 μL)	5	5	5	5	5	5	5
pre-enrichment (400 μL)	0.5	5	5	5	0.5	5	5

References

1. Storhoff, J. J., Elghanian, R., Mucic, R. C., Mirkin, C. A. & Letsinger, R. L. One-pot colorimetric differentiation of polynucleotides with single base imperfections using gold nanoparticle probes. *J Am Chem Soc* **120,** 1959-1964, https://doi.org/10.1021/ja972332i (1998).
2. Kor, K. et al. Structurally responsive oligonucleotide-based single-probe lateral-flow test for detection of miRNA-21 mimics. *Anal Bioanal Chem* **408,** 1475-1485, https://doi.org/10.1007/s00216-015-9250-9 (2016).
3. Karlsson, R., Michaelsson, A. & Mattsson, L. Kinetic analysis of monoclonal antibody-antigen interactions with a new biosensor based analytical system. *J Immunol. Methods* **145,** 229-240, https://doi.org/10.1016/0022-1759(91)90331-9 (1991).
4. Qian, S. & Bau, H. H. A mathematical model of lateral flow bioreactions applied to sandwich assays. *Anal Biochem* **322,** 89-98, https://doi.org/10.1016/j.ab.2003.07.011 (2003).
5. Han, C. M., KatiliTs, E. & Santiago, J. G. Increasing hybridization rate and sensitivity of DNA microarrays using isotachophoresis. *Lab Chip* **14,** 2958-2967, https://doi.org/10.1039/c41c00374h (2014).
6. Zhang, J. X. et al. Predicting DNA hybridization kinetics from sequence. *Nat Chem* **10,** 91-98, https://doi.org/10.1038/NCHEM.2877 (2018).
